# Congenital Synostosis of Cervical Vertebrae: An Osteological Study and Review of the Literature

**DOI:** 10.7759/cureus.6015

**Published:** 2019-10-28

**Authors:** George K Paraskevas, George Noussios, Konstantinos N Koutsouflianiotis, Kalliopi Iliou

**Affiliations:** 1 Orthopaedics, Aristotle University of Thessaloniki, Thessaloniki, GRC; 2 Physical Education and Sports Sciences, Aristotle University of Thessaloniki, Thessaloniki, GRC; 3 Internal Medicine, General Hospital of Thessaloniki "G. Gennimatas", Thessaloniki, GRC; 4 Psychiatry, Aristotle University of Thessaloniki, Thessaloniki, GRC

**Keywords:** axis, fusion, cervical vertebra, congenital synostosis

## Abstract

Introduction

The possible appearance of congenital fusion of the second cervical vertebra with adjacent cervical vertebrae, along with its epidemiology, embryological development, and clinical manifestations, was the aim of the current study.

Methods

The osteological material of 93 dried second cervical vertebrae of both sexes were examined in order to identify the likely presence of congenital fusion with the neighboring vertebrae.

Results

Among 93 axes, we identified one case of a congenitally fused second cervical vertebrae with the third and fourth cervical vertebrae, which accounted for a frequency of 1.08%. There was an incomplete fusion of the vertebral bodies and almost complete fusion of the laminae and facet joints.

Conclusion

The knowledge of such rare vertebral synostosis is crucial for the neurosurgeon, orthopedist, and physician dealing with the cervical spine, as well as the anesthetist when performing procedures, such as endotracheal intubation.

## Introduction

Among the significant variations of the cervical vertebrae is the occipitalization of the atlas and the fusion of the cervical vertebrae. The latter condition may be acquired, as occurs in tuberculosis, other infections, traumatic conditions, or juvenile rheumatoid arthritis [1], or of congenital origin. There are pathological conditions, such as diffuse idiopathic skeletal hyperostosis or ankylosing spondylosis, in which ossification of the paraspinal ligaments (as well as a fusion of the vertebral bodies) are occurring, chiefly in the thoracic region [2]. The congenital fused cervical vertebrae (CFCV), which are usually two and, rarely, more than two in number, constitute a solitary unit that functions as one vertebra [3]. The incidence of CFCV varies between 0.5% [4] and 6.25% [5], whereas the frequency for the fused second and third cervical vertebrae (C2-C3) varies between 0.10% [6] and 1.33% [7]. CFCV usually are asymptomatic until adulthood, when degenerative changes, such as disc hernias and arthritis, are occurring above and below the CFCV’s level; in those cases, it is likely symptoms of nerve root compression will be noticed [8].

In the current study, we detected the possible presence of congenitally fused C2 vertebra with the adjacent vertebrae, along with its morphological features, its clinical manifestations, embryological, and epidemiological aspects as well.

## Materials and methods

The current study was conducted on 93 Northern Greek dried adult vertebral columns derived from the osteological collection of the Department of Anatomy, Faculty of Medicine, Aristotle University of Thessaloniki, Greece. All the vertebrae were explored and examined analytically in order to find any possible abnormal fusion between neighboring vertebral bodies, laminae, transverse, or spinous processes. The CFCV were compared with the normal cervical vertebrae. Cases of broken, neonatal, damaged, or non-dried cervical vertebrae were excluded from the present research. The CFCV were photographed from different aspects with a digital camera Nikon D3400 (Nikon Corp., Tokyo, Japan).

## Results

**Figure 1:**
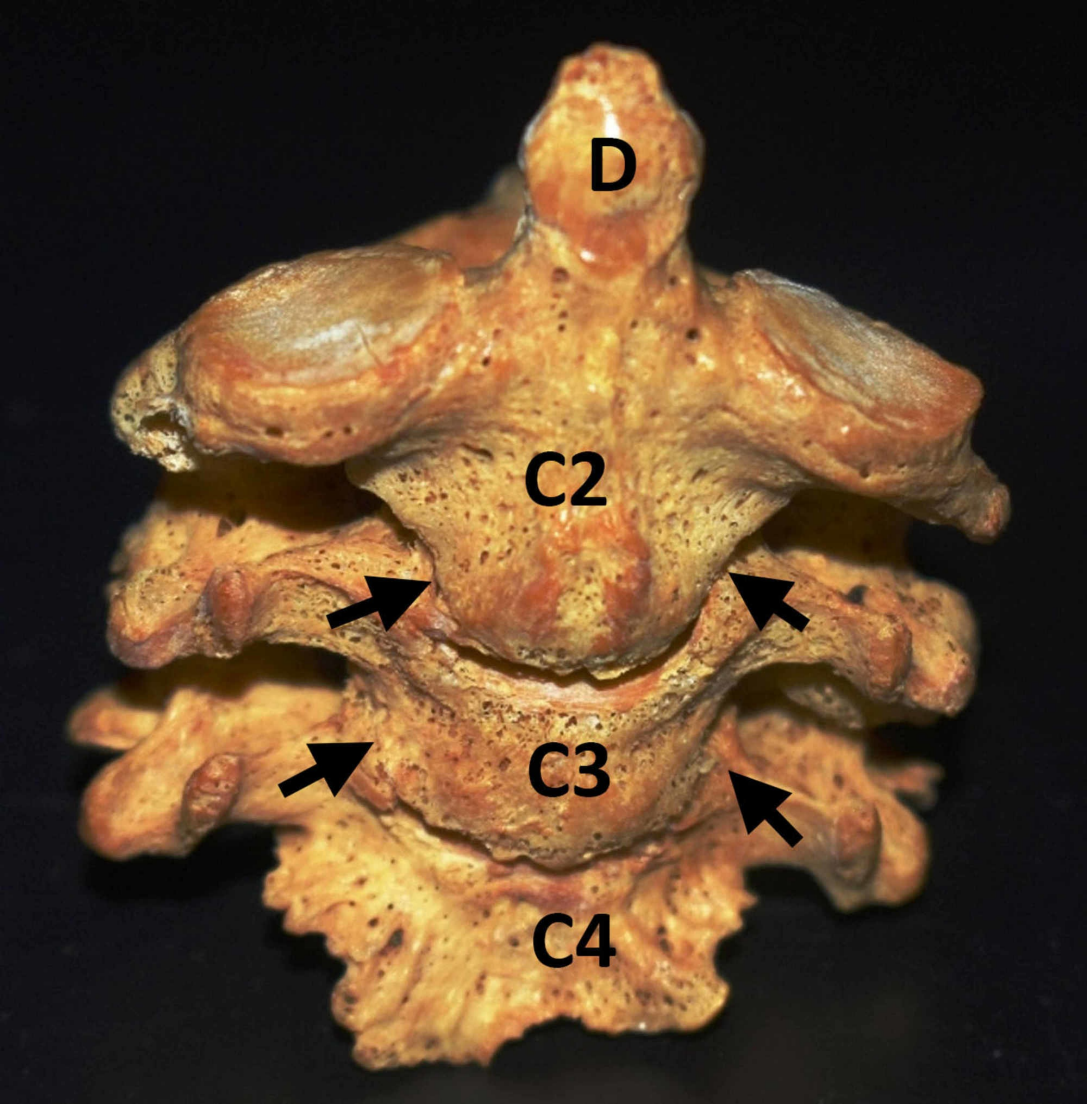
Anterior view of the fused C2-C4 Anterior view of the fused C2, C3, C4 (cervical vertebrae) where the vertebral bodies are seen partially fused at the areas of the uncovertebral joints (arrows); dens (D) of the axis.

**Figure 2:**
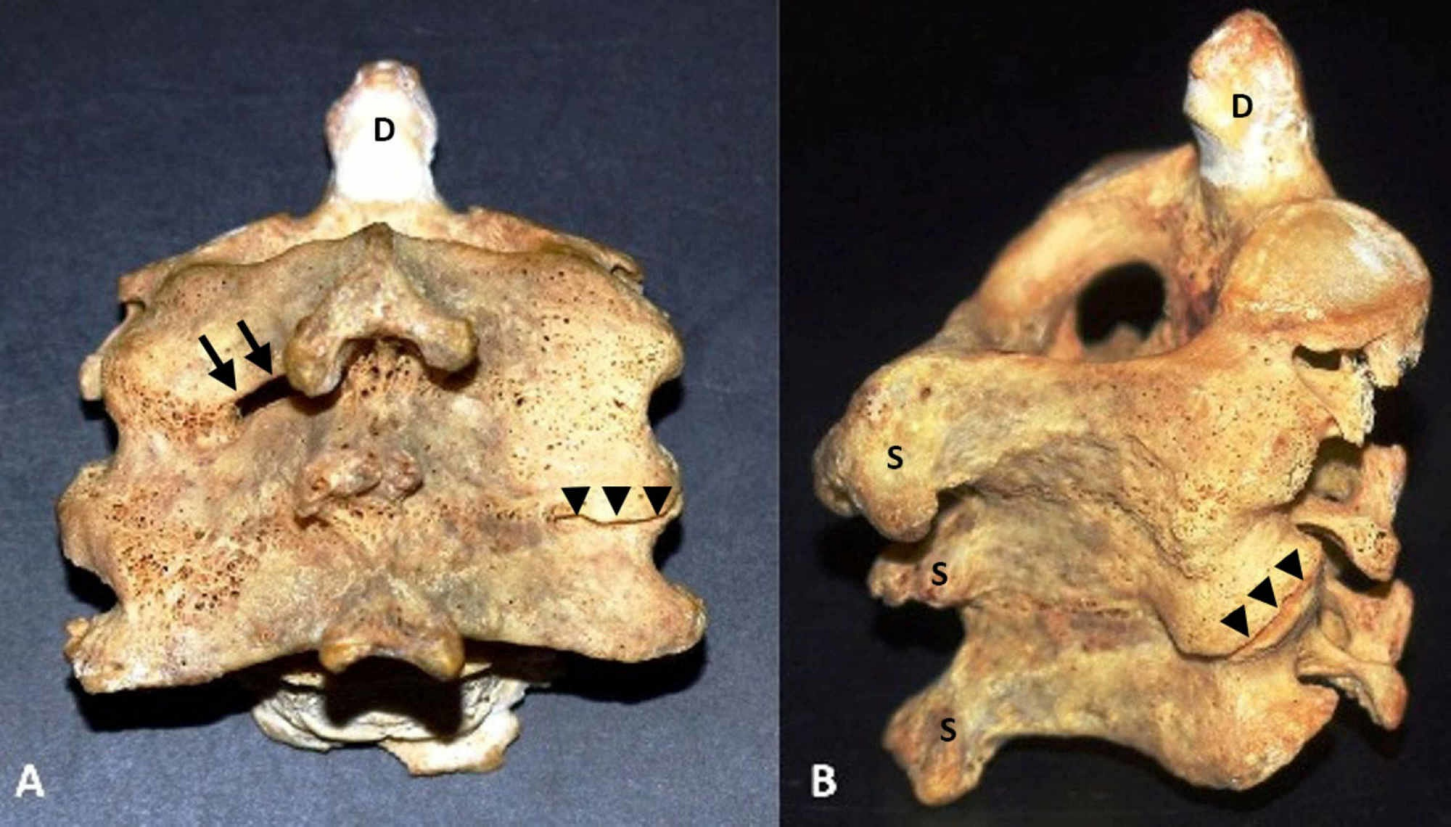
Posterior-inferior and right lateral aspect of the fused C2-C4 vertebrae Posterior-inferior (A) and right lateral (B) aspect of the fused C2-C4 vertebrae where the laminae are seen completely fused, except the left laminae C2 and C3 vertebrae which are unfused (arrows). The facet joints are also fused except for the right joint between C3 and C4 vertebrae (arrowheads). Furthermore, the spinous processes are shown being partially fused D: dens of the axis; S: spinous processes

Among the second cervical vertebrae (axis) derived from 93 different individuals from both genders from osteological material, we discovered one specimen of block vertebrae which involved the C2, C3, and C4 vertebrae, thus an incidence of 1.08%. Specifically, we observed the following: 1) The vertebral bodies of the three above-mentioned vertebrae were fused laterally at the sites of the uncovertebral joints (Figure 1); 2) Articular processes between the adjacent C2 - C4 vertebrae were completely fused, except the right zygapophysial joint between the C3 and C4 vertebrae; 3) The laminae of C2 - C4 vertebrae were completely fused, except the left laminae of C2 and C3 vertebrae which were unfused; 4) The spinous processes of C2 - C4 vertebrae were partially fused, whereas the transverse processes were not fused and the transverse foramina, as well as the intervertebral foramina, were clearly seen on both sides (Figure 2).

## Discussion

The CFCV may be distinguished from the acquired synostosis that is due to several causes, such as tuberculosis, other infections, juvenile rheumatoid arthritis, and trauma [1]. In CFCV, two (and rarely, more than two) cervical vertebrae are usually fused, appearing not only structurally as one but also functioning as one [3]. The CFCV can be distinguished by the acquired fused cervical vertebrae by two factors: one factor is a decrease in the sagittal diameter of the vertebrae, and the second factor is that the total height of the fused vertebrae is equal to the fused vertebrae’s height, including the intervertebral disc [9-11]. On the contrary, Soni et al. claimed that in the congenital fusion of the cervical region, there is a decrease in spine length [12]. Brown et al. supported that in CFCV, the intervertebral discs are absent, the transverse foramina are smooth, the vertebral body height is maintained, and a single spinous process exists for two vertebral bodies [1]. Furthermore, Klosinski et al. mentioned that in the Klippel-Feil syndrome, the CFCV are altered and display a grossly abnormal appearance [13]. 

The CFCV may be clinically asymptomatic and silent and are often detected by an orthopedist, neurosurgeon, general practitioner, or orthodontist as incidental findings during a radiologic examination. Usually, CFCV are asymptomatic until young adulthood. CFCV, in cases of degenerative changes of the upper and lower portion of the cervical spine and the presence of osteophytes and intervertebral disc tears at the adjacent regions, may induce symptoms and signs of nerve compression. Nerve root compression may occur in cases with the narrow intervertebral foramina of a CFCV leading likely to sensory and motor disturbances of the concerned parts of the body [8]. In advanced age, CFCV result in hypermobility and great biomechanical stress leading to degenerative arthritis above and below them. Moreover, minor but intermittent head and neck pain are noted, the trapezii muscles are prominent, the neck movements are restricted, and the hairline is lowered [7, 11-12]. Furthermore, in cases of a pronounced asymmetry between the two halves of the vertebral body of a CFCV, torticollis will likely develop with a markedly titled position of the head [14]. Klosinski et al. noted that in atlantoaxial synostosis, the vertebral vessels could be potentially compressed, leading to symptoms like occipital and neck pain, vertigo, neurological deficits, and presumably cerebral ischemic infarction, especially in atlantoaxial synostosis where the head rotation is seriously limited [13].

In severe cases, CFCV may induce myelopathy presumably due to spinal stenosis. In some cases, it has been reported that intervertebral disc tear, rupture of the transverse ligament of the atlas, fracture of the odontoid process of the axis, and spondylolysis have been found [11, 15-16]. The presence of CFCV may likely cause the laxity of the ligaments between the occiput and the atlas, leading to the brainstem or spinal cord compression with associated symptoms [17]. Major neurological complications involve quadriplegia or death after minor trauma in high-risk patients [18]. CFCV may be associated with a syndrome known as Klippel-Feil syndrome, which is a complex congenital disease with various clinical manifestations [14]. In 1919, Feil subdivided that syndrome into Type 1 with massive fusion of many cervical and upper thoracic vertebrae into blocks, Type 2 with fusion of only one or two interspaces (usually C2-C3 or C5-C6), and Type 3 with both cervical fusion and lower thoracic or lumbar fusion often associated with multiple organ abnormalities and neurological compromise [19]. Furthermore, CFCV may be noticed in Wildervanck syndrome, which is characterized by a Klippel-Feil deformity of the cervical vertebrae in combination with abducens palsy with retracted bulbi and hearing loss [20].

According to Soni et al., the decreasing order of frequency is C2-C3, C5-C6, L4-L5, and any section of the thoracic spine [12]. Sharma et al. studied 48 dried adult vertebral columns and noted CFCV in 6.25%, fused thoracic vertebrae in 4.16%, and fused lumbar vertebrae in 2.08% [5]. Nazeer et al. examined 2,400 ossified dried vertebrae and encountered CFCV in 0.5%, fused thoracic vertebrae in 0.08%, and fused lumbar vertebrae in 0% [4], whereas, according to Deepa et al. who studied 50 dry adult vertebral columns, the above-mentioned incidences were 2%, 4%, and 2%, respectively [21]. Ajay et al. found CFCV incidence of 1.4% in the material of 280 dried adult cervical vertebrae [22]. The fusion of thoracic vertebrae is usually associated with ossification of an anterior longitudinal ligament in diffuse idiopathic skeletal hyperostosis or ankylosing spondylitis [2]. Soni et al. reported for the fused C2-C3 an incidence of 0.4% to 0.7% with no sex predilection [12]. Shands et al. found an incidence of fused C2-C3 (0.5%) in the radiographic material of 700 patients [23], whilst Kadadi et al. found one incidence of a fused C2-C3 (1.33%) in their review of 75 C2 vertebrae [7]. Ajay et al. mentioned an incidence of 0.36% for the fused C2-C3 [22], whereas Roy et al. found a frequency of 0.10% [6]. It has been noted that up to 70% of atlantooccipital fusions have an associated C2-C3 fusion with instability at the atlantoaxial joint [12].

As per the embryological development of CFCV, it is considered that these anomalies are due to malformations of the notochord, associated especially with defects of the cervical somites [24]. It is believed that there is a disturbance of the normal spinal subdivision due to an inadequate blood supply during the third to eighth week of fetal development [25]. In particular, a combination of genetics and environment occurs during the third week of embryologic development leading to CFCV formation [26]. In cases of acquired CFCV, it is considered that even a microtrauma activates local factors, such as bone morphogenic proteins or prostaglandins, that induce modification of mesenchymal cells to osteoblasts. Moreover, after a neurologic lesion, the joint proprioception is disrupted, leading to heterotopic ossification [27].

The knowledge of a likely presence of CFCV is essential for the patient since he/she has to modify his/her lifestyle and avoid possible traumatic injuries of the head and neck, as well as extensive manipulations of rotation and extension of the head that may presumably induce compression of the spinal cord and/or vertebral artery [12]. The possible appearance of CFCV may cause problems in the determination of the vertebral level during neurosurgical procedures in the cervical region [28]. Moreover, the hyperextension of the neck during anesthetic procedures, such as endotracheal intubation, can predispose to intervertebral disc prolapse in individuals with CFCV. The anesthesiologist should also take into account the possible presence of CFCV when performing a cisternal puncture [29]. Ultimately, the awareness of CFCV’s existence may assist the identification of dead bodies by taking antemortem radiographs [5].

## Conclusions

The congenital synostosis of cervical vertebrae is a rare condition (incidence of 1.08% in the Northern Greek population), but when it is present, symptoms of nerve roots compression are noticed. The pathogenetic pathway begins during the embryological development of the cervical vertebrae, and when it is considered an acquired CFCV, usually a microtrauma triggers local factors leading to heterotopic ossification. The knowledge of such a condition is significant for the patient in order to modify his/her lifestyle and avoid injuries of the neck, but also for the neurosurgeon and anesthesiologist in order to prevent possible complication during surgeries in the region. 
